# Association between Preoperative C-Reactive Protein-to-Albumin Ratio and Mortality after Plastic and Reconstructive Surgery

**DOI:** 10.3390/jcm13071998

**Published:** 2024-03-29

**Authors:** Ah Ran Oh, Ha Min Sung, Jungchan Park, Gayoung Jin, So Myung Kong, Minsu Jung, Sangmin Maria Lee

**Affiliations:** 1 Department of Anesthesiology and Pain Medicine, Samsung Medical Center, Sungkyunkwan University School of Medicine, Seoul 06351, Republic of Korea; aran8612@gmail.com (A.R.O.); gayoung13.jin@samsing.com (G.J.); somyung.kong@samsung.com (S.M.K.); maria3run@gmail.com (S.M.L.); 2 Department of Anesthesiology and Pain Medicine, Kangwon National University Hospital, Chuncheon 24341, Republic of Korea; 3 Link Plastic Surgery Clinic, Seoul 06120, Republic of Korea; brismar@naver.com (H.M.S.); minsuj83@naver.com (M.J.)

**Keywords:** biomarkers, mortality, plastic surgery

## Abstract

**Background**: Prognostic markers have not been extensively studied in plastic and reconstructive surgery. Objective: We aimed to evaluate the prognostic value of preoperative C-reactive protein (CRP)-to-albumin ratio (CAR) in plastic and reconstructive surgery and to compare it with the neutrophil-to-lymphocyte ratio (NLR), platelet-to-lymphocyte ratio (PLR), and modified Glasgow prognostic score (mGPS). **Methods**: From January 2011 to July 2019, we identified 2519 consecutive adult patients who were undergoing plastic and reconstructive surgery with available preoperative CRP and albumin levels. The receiver operating characteristic (ROC) curve was generated to evaluate predictability and estimate the threshold. The patients were divided according to this threshold, and the risk was compared. The primary outcome was one-year mortality, and the overall mortality was also analyzed. **Results**: The one-year mortality was 4.9%. The CAR showed an area under the ROC curve of 0.803, which was higher than those of NLR, PLR, and mGPS. According to the estimated threshold of 1.05, the patients were divided into two groups; 1585 (62.9%) were placed in the low group, and 934 (37.1%) were placed in the high group. After inverse probability weighting, the mortality rate during the first year after plastic and reconstructive surgery was significantly increased in the high group (1.3% vs. 10.9%; hazard ratio, 2.88; 95% confidence interval, 2.17–3.83; *p* < 0.001). **Conclusions**: In this study, high CAR was significantly associated with one-year mortality of patients after plastic and reconstructive surgery. Further studies are needed on prognostic markers in plastic and reconstructive surgery.

## 1. Introduction

Plastic and reconstructive surgery involves many applications and complex procedures. However, these surgeries are generally considered relatively safe, with a low risk of death [1]. Improvements in surgical and anesthetic techniques have increased the safety of plastic and reconstructive surgery. As a result, deaths from these procedures are rare; however, when they do occur, they can be costly. While the risks associated with other types of major surgery have been thoroughly studied, the risk factors for perioperative mortality in plastic and reconstructive surgery have not been as extensively researched [2].

Several simplified markers have been proposed for predicting prognosis, including biologic markers, such as C-reactive protein (CRP)-to-albumin ratio (CAR), neutrophil-to-lymphocyte ratio (NLR), platelet-to-lymphocyte ratio (PLR), and modified Glasgow prognostic score (mGPS) [3,4,5,6,7]. In fact, NLR has been significantly associated with mortality following plastic and reconstructive surgery in our previous study [8]. Recently, increasing evidence has indicated that combining markers of inflammatory response with nutritional condition could better reflect clinical prognosis. In particular, the CAR has been demonstrated as a prognostic marker of various clinical situations, but it has not been validated in plastic and reconstructive surgery [7]. Therefore, we enrolled patients undergoing plastic and reconstructive surgery with available preoperative CRP and albumin levels and evaluated whether preoperative CAR was associated with postoperative mortality. We also compared the predictive value with biomarkers of NLR, PLR, and mGPS. Our findings may help clinicians to predict postoperative outcomes using a simple index.

## 2. Materials and Methods

This study was a retrospective observational study that was approved by the Institutional Review Board at Samsung Medical Center (SMC 2020-09-001). The requirement for written informed consent was waived by the Institutional Review Board at Samsung Medical Center because we extracted the data in de-identified form. We conducted this research following the Declaration of Helsinki and reported the results according to the Strengthening the Reporting of Observational Studies in Epidemiology guidelines.

### 2.1. Study Population and Data Collection

We enrolled consecutive adult patients who underwent plastic and reconstructive surgery at Samsung Medical Center between January 2011 and July 2019. We excluded patients without preoperative CRP and albumin levels. The data were extracted in a de-identified form from our electronic archive system, Clinical Data Warehouse Darwin-C. This electronic system allows researchers to review and retrieve data for investigation from electronic hospital records and contains more than 2.2 million surgeries, 1 billion laboratory findings, 100 million disease codes, and 200 million prescriptions. The results of blood laboratory tests were automatically extracted for analysis. In this study, the mortality data from other hospitals were regularly checked and updated using the National Population Registry of the Korea National Statistical Office, so there was no missing information on deaths. The medical records were reviewed by investigators who were blind to patient mortality to avoid bias.

### 2.2. Study Outcomes and Definitions

The primary endpoint in this study was mortality during a one-year follow-up period that began immediately after surgery. The secondary outcome was mortality during the overall follow-up period. The Charlson Comorbidity Index was calculated using the 10th revision of the International Statistical Classification of Diseases and Related Health Problems (ICD-10) [9].

According to our institutional protocol, patients exhibiting clinical symptoms suggestive of infection undergo a comprehensive evaluation, including blood laboratory tests, before surgery. The attending clinician assesses the risks and benefits of surgical procedures, and at their discretion, patients may be referred to the Department of Infectious Diseases for further evaluation.

### 2.3. Statistical Analysis

The baseline characteristics are described using the mean (±standard deviation) or median (interquartile range) for continuous variables and the number and percentage of categorical variables. To compare differences between the two groups, the *t*-test or Mann–Whitney U test was used for continuous variables, and the chi-square or Fisher’s exact test was employed for categorical variables. To evaluate the predictability of CAR, NLR, and PLR results and their association with one-year mortality, receiver operating characteristics (ROC) curve analysis was performed, and Youden’s Index was used to estimate the optimal cut-off. We compared the predictability using the comparison between the area under the ROC curve (AUC) using a nonparametric approach [10]. The predictabilities were also evaluated according to surgery type and presence of malignancy. Based on the estimated cut-off value of CAR, the study patients were classified into either a low or a high group. The mortalities were compared with Cox regression analysis. To control for selection bias and other confounding factors, we adjusted for differences in baseline patient characteristics using inverse probability weighting (IPW). This technique involves the use of weights that are the reciprocal of the propensity scores to balance the groups. A difference <10% in absolute standardized difference (ASD) between the groups was considered a balanced comparison [11]. The differences in mortality were reported as hazard ratios (HRs) and 95% confidence intervals (CIs) using Cox proportional hazards analysis with IPW. The power of analysis was evaluated based on sample size [12]. Kaplan–Meier estimates were used to create survival curves, which were compared using the log-rank test. All statistical analyses were performed using R 4.0.2 (Vienna, Austria; http://www.R-project.org/, accessed on 27 February 2024). All tests were two-tailed, and *p* < 0.05 was considered statistically significant.

## 3. Results

Of the 7089 patients who underwent plastic and reconstructive surgery from January 2011 to July 2019 at our institution, 2519 patients with available preoperative CRP and albumin levels were enrolled for analysis. The mean values of CRP were 1.33 for head and neck surgery, 1.18 for trunk and extremities surgery, 1.42 for breast surgery, 1.21 for aesthetic surgery, and 1.32 for mass excision surgery. We divided the study patients into survivors and non-survivors during the one-year follow-up period after surgery. The median follow-up period for one-year mortality was 365 (365–365) days, and the median overall follow-up period was 1055 (371–2029) days. The one-year mortality was 4.9%, and the baseline characteristics according to one-year mortality are summarized in Table 1. The median value of CAR was much higher in the non-survivor group (0.38 vs. 7.86), as were NLR (1.93 vs. 4.56) and PLR (7.90 vs. 11.17), respectively.

**Figure 1:**
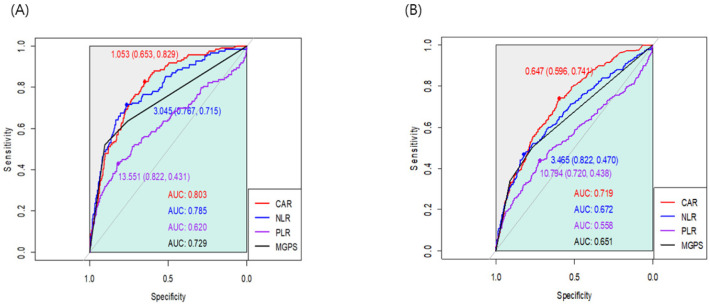
Receiver operating characteristic curves for the associations of CAR, NLR, PLR, and mGPS with (**A**) one-year mortality and (**B**) overall mortality.

**Figure 2:**
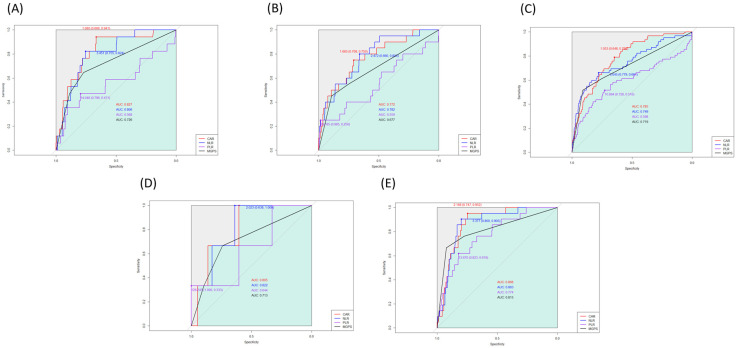
Receiver operating characteristic curves for the associations of CAR with one-year mortality in (**A**) head and neck, (**B**) trunk and extremities, (**C**) breast, (**D**) aesthetic surgery, and (**E**) mass excision.

We generated the ROC curve for each index, and the AUC of CAR, NLR, PLR, and mGPS were 0.803, 0.785, 0.620, and 0.729, respectively (Figure 1). In the AUC comparison, CAR showed no significant difference from NLR (z = 0.84; *p* = 0.401). Based on the maximum Youden’s Index, the optimal cut-off threshold values of CAR, NLR, and PLR were 1.05, 3.04, and 13.55 for one-year mortality, respectively. The ROC curves were also generated for overall mortality (Figure 1), and the AUC of CAR was significantly higher than that of NLR (0.719 vs. 0.672; z = 2.45; *p* = 0.01). In the subgroup analysis according to surgery type, the AUC of CAR was 0.827 for head and neck surgery, 0.772 for trunk and extremities surgery, 0.785 for breast surgery, 0.805 for aesthetic surgery, and 0.868 for mass excision surgery. The estimated thresholds were 1.08, 1.69, 1.05, 0.81, and 0.87, respectively (Figure 2). In patients without malignancy, the AUC of CAR, NLR, PLR, and mGPS were 0.819, 0.794, 0.617, and 0.745, respectively.

**Figure 3:**
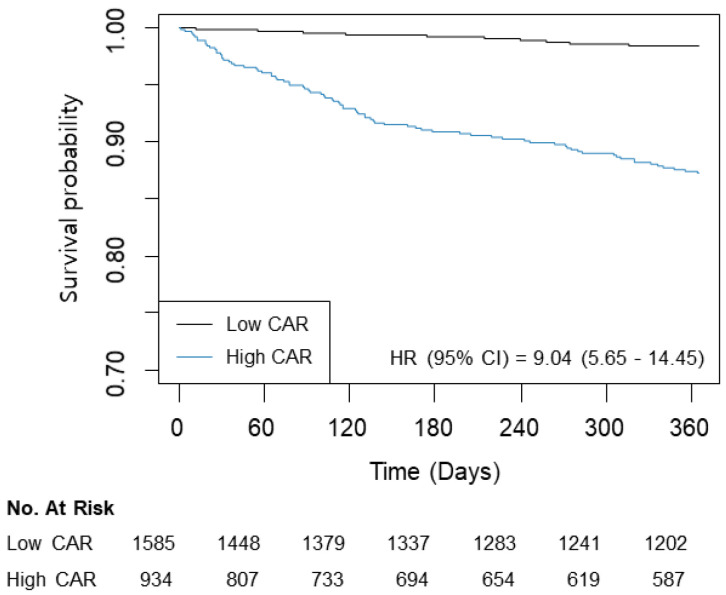
Kaplan–Meier curves for one-year mortality according to CAR (≤1.05/>1.05).

The patients were divided according to the estimated threshold of CAR associated with one-year mortality. Using a CAR of 1.05, 1585 (62.9%) patients were classified into the low group, and 934 (37.1%) were placed into the high group (Table 2). The high group tended to be older with more habitual risk factors. The incidence rates of the underlying disease and the Charlson Comorbidity Index were also higher in the high group. After an adjustment using an IPW technique, ASD < 10% was suggested to offer a successful balance of all covariates between the groups. The unadjusted analysis showed an increased incidence and risk of mortality in the high group for one year (1.3% vs. 10.9%; HR: 9.04; 95% CI: 5.65–14.45; *p* < 0.001) and overall (5.4% vs. 17.7%; HR: 3.97; 95% CI: 3.06–5.16; *p* < 0.001) follow-up periods (Table 3). An IPW analysis revealed similar results (HR: 2.88; 95% CI: 2.17–3.83; *p* < 0.001 for one year and HR, 5.48; 95% CI: 3.33–9.03; *p* < 0.001 for overall mortality). The survival curves are presented in Figure 3. Based on the sample size, the power of analysis was 0.99 when HR was >1.2.

## 4. Discussion

The main finding of this study was that preoperative CAR was associated with increased mortality. The estimated threshold of CAR was 1.05 for one-year mortality, and the predictability was comparable with that of NLR. For overall mortality, the AUC of CAR was even higher than that of NLR. Our results suggest that CAR before plastic and reconstructive surgery may be used as a simple biomarker of mortality that is readily available and relatively inexpensive.

Plastic and reconstructive surgeries involve a wide range of procedures and body areas, but patients undergoing these surgeries are generally healthier compared to those who undergo other types of major surgeries. Therefore, not much attention has been given to predicting the risks for patients based on preoperative conditions. Recently, several biomarkers that combine various laboratory tests have been proposed to predict the prognosis of patients preoperatively [3,4,5,6,7]. Our previous study also indicated that NLR was associated with postoperative mortality in patients undergoing plastic and reconstructive surgery [8], but various biomarkers have not been thoroughly studied. 

In this study, CAR showed a good predictability for mortality during the one-year follow-up after surgery. CAR is a new and promising biomarker that is determined by the ratio of CRP and albumin levels in the blood. It is a combination of inflammatory- or nutrition-related markers, which have been widely evaluated in various clinical situations [3,7,13,14]. The presence of inflammation and nutritional impairment are well-known risk factors for poor prognosis. The presence of infection, comorbidities, and malnutrition are shown to induce inflammation and deterioration of a patient’s nutritional status [15,16,17], and this is likely to also be found in patients anticipating a scheduled surgery. In surgical patients, pre-existing inflammation can worsen malnutrition and increase the inflammatory response to surgical injuries [18], which can have a significant impact on mortality. 

Another notable finding was that CAR showed a significantly higher AUC for overall mortality compared with NLR, which suggests better predictability of a longer follow-up period. By combining CRP with albumin level, CAR reflects both the inflammatory and nutritional status of patients prior to surgery. A reflection of nutritional status may explain why CAR had a greater predictive value compared to other biomarkers, particularly for a long-term follow-up period. In addition, the time required to produce a noticeable decrease in nutritional status may be enough to have a significant effect on patient recovery. Other factors, such as the patient’s comorbidities and response to treatment, can play a more decisive role in their short-term outcome. The estimated thresholds for CAR varied significantly depending on the duration of the follow-up period. Further research is needed before CAR can be adopted into daily practice. The goal of our analysis is not to establish superiority among these variables in predicting mortality. Instead, we emphasize that CAR is independently associated with postoperative mortality. It may prove particularly helpful in clinical situations where the risk is not well-reflected in patient age or comorbidity.

The versatility of the CAR in predicting outcomes in plastic and reconstructive surgery is a noteworthy aspect highlighted in our study. Our subgroup analysis reveals an AUC across a diverse spectrum of surgical procedures. This is particularly significant in plastic and reconstructive surgery, where patients undergo various interventions in diverse medical conditions. Notably, almost half of the patients experiencing one-year mortality underwent wound debridement, indicating a likelihood of infectious complications. This suggests that CAR could serve as a valuable predictor of mortality, offering insights less influenced by age or chronic conditions. Conversely, in aesthetic procedures, more than one-third of patients exhibited elevated CAR, yet only three cases resulted in one-year mortality. This distinction underscores the need for a nuanced discussion regarding the safety of elective aesthetic surgeries. Our analysis identified specific thresholds for CAR based on surgery type, emphasizing the necessity for future studies to establish tailored thresholds for different surgical interventions.

The advantage of CAR in plastic and reconstructive surgeries shown in this study was that it could be used regardless of a wide range of surgical procedures. Our subgroup analysis demonstrated the AUC that suggests that CAR could be used to predict mortality regardless of surgery type. This indeed is a notable finding especially in plastic and reconstructive surgery, because it covers a wide variety of surgical procedures in patients with various medical conditions. In our analysis, almost half of patients with mortality showed one-year mortality. Most of these patients underwent wound debridement and were likely to be infectious. This suggests that CAR could be useful in predicting mortality that is less affected by age or chronic conditions. On the other hand, more than 1/3 of aesthetic patients showed high CAR, while only three patients showed one-year mortality. Moreover, the estimated threshold for CAR was different according to surgery type. So, future studies may need to establish different thresholds for more detailed surgery types.

The mGPS scoring system uses the same laboratory variables as CAR. The difference is that the mGPS assigns patients to a certain category rather than providing a specific value. The system is simple, and the score is considered one of the most effective biomarkers for predicting patient prognosis. Several studies have demonstrated similar prognostic values and performance for mGPS and CAR, particularly in patients with cancer [19,20]. However, our results indicated that CAR had better predictive value than mGPS. This finding may be related to the mGPS score of 0 of most of our study patients. Since the CAR can further stratify patients using a continuous numerical value, it may be a more sensitive marker in relatively healthier populations, such as ours. More research is needed to better understand the relationships between inflammation and nutrition-based markers and the mortality of surgical patients.

There are a few limitations that should be taken into account when interpreting the results of our study. First, it was a retrospective single-center study, so our results may have been influenced by factors that we were unable to control. Although we conducted statistical adjustments, the effects of unknown variables could not be taken into account. Second, preoperative CRP and albumin levels were selectively measured, which could have caused bias. In addition, due to the long study period, changes in surgical techniques and postoperative care may have affected the results. Third, different thresholds were estimated according to surgery type. Considering the wide variety of plastic and reconstructive surgical procedures, we acknowledge that more detailed analyses of specific surgery types may be warranted in future studies to provide a comprehensive understanding. Lastly, our study does not suggest a treatment plan for patients with elevated biomarkers. As a result, the optimal threshold cannot be established from our analysis. Further research in the form of a prospective randomized trial is needed. Despite these limitations, this is the first study to thoroughly examine biomarkers regarding their association with mortality after plastic and reconstructive surgery. 

In conclusion, a high CAR value was significantly associated with one-year patient mortality after plastic and reconstructive surgery. Further studies are necessary to explore other potential prognostic markers in plastic and reconstructive surgery. 

## Figures and Tables

**Table 1:** Baseline characteristics according to one-year mortality.

	Survivor (*N* = 2396)	Non-Survivor (*N* = 123)	*p*-Value
C-reactive protein/albumin ratio	0.38 (0.10–2.46)	7.86 (1.93–18.02)	<0.001
Neutrophil/lymphocyte ratio	1.93 (1.37–2.94)	4.56 (2.51–8.17)	<0.001
Platelet/lymphocyte ratio	7.90 (5.79–11.59)	11.17 (6.41–23.79)	<0.001
Modified Glasgow prognostic score			<0.001
0	1818 (75.9)	45 (36.6)	
1	352 (14.7)	14 (11.4)	
2	226 (9.4)	64 (52.0)	
C-reactive protein, mg/L	1.60 (0.40–9.30)	27.30 (6.30–52.60)	<0.001
Albumin, g/dL	4.30 (3.90–4.60)	3.10 (2.75–3.60)	<0.001
Neutrophil	58.8 (51.1–67.0)	72.6 (62.0–81.2)	<0.001
Lymphocyte	30.6 (22.7–37.6)	15.2 (10.0–24.9)	<0.001
Platelet, K/mcL	239 (198–258)	199 (111–285)	<0.001
Age, years	51 (40–61)	65 (52–74)	<0.001
Male	1012 (42.2)	64 (25.0)	0.04
Smoking	342 (14.3)	19 (15.4)	0.82
Alcohol	635 (26.5)	22 (17.9)	0.04
Hypertension	328 (13.7)	22 (17.9)	0.24
Charlson comorbidity index	0.60 (±1.39)	1.29 (±1.85)	<0.001
*Myocardial infarction*	9 (0.4)	3 (2.4)	
*Heart failure*	19 (0.8)	5 (4.1)	
*Peripheral vascular disease*	15 (0.6)	3 (2.4)	
*Cerebrovascular disease*	88 (3.7)	5 (4.1)	
*Dementia*	0	1 (0.8)	
*Chronic pulmonary disease*	4 (0.2)	0	
*Rheumatic disease*	55 (2.3)	5 (4.1)	
*Peptic ulcer disease*	0	0	
*Mild liver disease*	160 (6.7)	18 (14.6)	
*Diabetes without complication*	210 (8.8)	21 (17.1)	
*Diabetes with complication*	93 (3.9)	10 (8.1)	
*Hemiplegia*	19 (0.8)	1 (0.8)	
*Renal disease*	91 (3.8)	14 (11.4)	
*Any malignancy*	93 (3.9)	7 (5.7)	
*Moderate to severe liver disease*	5 (0.2)	0	
*Metastatic solid tumor*	0	0	
*Human immunodeficiency virus*	0	1 (0.8)	
Preoperative blood test			
Hemoglobin, g/dL	13.1 (±1.8)	12.9 (±1.9)	0.4
Creatinine, mg/dL	0.90 (±0.87)	1.15 (±1.36)	0.003
Operative variables			
Duration, hours	2.48 (0.98–4.59)	1.00 (0.54–3.40)	<0.001
General anesthesia	2007 (83.8)	63 (51.2)	<0.001
Emergency surgery	239 (10.0)	33 (26.8)	<0.001
Type			0.66
*Head and neck*	383 (16.0)	17 (13.8)	
*Trunk and extremities*	476 (19.9)	20 (16.3)	
*Breast*	1044 (43.6)	62 (50.4)	
*Aesthetic surgery*	58 (2.4)	3 (2.4)	
*Mass excision*	435 (18.2)	21 (17.1)	

Values are *n* (%), mean (±standardized difference), or median (interquartile range). Abbreviations: ASD, absolute standardized mean difference; IPW, inverse probability weighting.

**Table 2:** Baseline characteristics according to the estimated cut-off point of C-reactive protein/albumin ratio > 1.05.

	Low Group (*N* = 1585)	High Group (*N* = 934)	*p*-Value	ASD before IPW	ASD after IPW
C-reactive protein/albumin ratio	0.14 (0.07–0.36)	5.00 (2.33–12.34)	<0.001		
Neutrophil/lymphocyte ratio	1.74 (1.28–2.47)	2.65 (1.75–4.56)	<0.001		
Platelet/lymphocyte ratio	7.28 (5.56–9.76)	10.51 (6.84–16.67)	<0.001		
Modified Glasgow prognostic score			<0.001		
0	1585 (100)	278 (29.8)			
1	0	366 (39.2)			
2	0	290 (31.0)			
C-reactive protein, mg/L	0.60 (0.30–1.50)	18.25 (8.90–45.25)	<0.001		
Albumin, g/dL	4.40 (4.20–4.70)	3.70 (3.20–4.30)	<0.001		
Neutrophil	56.9 (50.2–64.1)	64.3 (55.3–72.9)	<0.001		
Lymphocyte	32.6 (26.0–39.3)	24.0 (16.0–31.9)	<0.001		
Platelet, K/mcL	236 (199–273)	245 (188–316)	<0.001		
* Age, years	51 (41–59)	55 (39–67)	<0.001	19.3	0.5
* Male	621 (39.2)	455 (48.7)	0.04	19.3	1.5
* Smoking	206 (13.0)	155 (16.6)	0.02	10.1	5.2
* Alcohol	442 (27.9)	215 (23.0)	0.01	11.2	6.5
* Hypertension	195 (12.3)	155 (16.6)	0.003	12.2	2
* Charlson comorbidity index	0.53 (±1.24)	0.85 (±1.68)	<0.001	21.8	0.7
*Myocardial infarction*	4 (0.3)	8 (0.9)			
*Heart failure*	6 (0.4)	18 (1.9)			
*Peripheral vascular disease*	6 (0.4)	12 (1.3)			
*Cerebrovascular disease*	57 (3.6)	36 (3.9)			
*Dementia*	0	1 (0.1)			
*Chronic pulmonary disease*	1 (0.1)	3 (0.3)			
*Rheumatic disease*	23 (1.5)	37 (4.0)			
*Peptic ulcer disease*	0	0			
*Mild liver disease*	103 (6.5)	75 (8.0)			
*Diabetes without complication*	119 (7.5)	112 (12.0)			
*Diabetes with complication*	47 (3.0)	56 (6.0)			
*Hemiplegia*	9 (0.6)	11 (1.2)			
*Renal disease*	34 (2.1)	71 (7.6)			
*Any malignancy*	74 (4.7)	26 (2.8)			
*Moderate to severe liver disease*	1 (0.1)	4 (0.4)			
*Metastatic solid tumor*	0	0			
*Human immunodeficiency virus*	0	1 (0.1)			
Preoperative blood test					
* Hemoglobin, g/dL	13.1 (±1.8)	13.0 (±1.8)	0.24	4.9	3.4
* Creatinine, mg/dL	0.81 (±0.38)	1.07 (±1.37)	<0.001	26	0.8
Operative variables					
* Duration, hours	2.78 (1.10–4.77)	1.72 (0.70–4.20)	<0.001	23.3	1.2
* General anesthesia	1428 (90.1)	642 (68.7)	<0.001	54.8	3.8
* Emergency surgery	97 (6.1)	175 (18.7)	<0.001	39	1.6
* Type			0.96	3.2	6.4
*Head and neck*	255 (16.1)	145 (15.5)			
*Trunk and extremities*	309 (19.5)	187 (20.0)			
*Breast*	690 (43.5)	416 (44.5)			
*Aesthetic surgery*	39 (2.5)	22 (2.4)			
*Mass excision*	292 (18.4)	164 (17.6)			

Values are *n* (%), mean (±standardized difference), or median (interquartile range). Abbreviations: ASD, absolute standardized mean difference; IPW, inverse probability weighting. * The following variables were retained for IPW adjustment.

**Table 3:** Mortalities according to the estimated cut-off point of C-reactive protein/albumin ratio > 1.05.

	Low Group (*N* = 1585)	High Group (*N* = 934)	Unadjusted HR (95% CI)	*p*-Value	IPW Adjusted HR	*p*-Value
One-year mortality	21 (1.3)	102 (10.9)	9.04 (5.65–14.45)	<0.001	2.88 (2.17–3.83)	<0.001
Overall mortality	86 (5.4)	165 (17.7)	3.97 (3.06–5.16)	<0.001	5.48 (3.33–9.03)	<0.001

Abbreviations: IPW, inverse probability weighting; HR, hazard ratio; CI, confidence interval. IPW adjustment analysis retained age, male, hypertension, smoking, alcohol, Charlson comorbidity index, preoperative creatinine and hemoglobin levels, operative duration, general anesthesia, and type of surgery.

## Data Availability

All relevant data are provided within the paper.
